# Supplementary material for: Difficult-to-treat resistant Pseudomonas aeruginosa infections in Lebanese hospitals: Impact on mortality and the role of initial antibiotic therapy
Source: PLoS One. 2025 May 12;20(5):e0321935. doi: 10.1371/journal.pone.0321935 (PMC12068612; doi:10.1371/journal.pone.0321935)
Supplement: S1 Table — (DOCX) [file pone.0321935.s001.docx]

**S1 Table**. *In vitro* antimicrobial susceptibility testing data for *Pseudomonas aeruginosa* isolates (N = 477).

| **Antibiotic Class** | **Antibiotics** | **Isolates^a^**  **(n)** | **Sensitive**  **n (%)** | **Resistant**  **n (%)** | **Intermediate**  **n (%)** |
| --- | --- | --- | --- | --- | --- |
| **Penicillins** | Piperacillin | 313 | 214 (68.4) | 59 (18.8) | 40 (12.8) |
|  | Piperacillin-tazobactam | 471 | 340 (72.2) | 77 (16.3) | 54 (11.5) |
|  | Ticarcillin | 199 | 104 (52.3) | 65 (32.7) | 30 (15.1) |
|  | Ticarcillin-clavulanate | 211 | 108 (51.2) | 63 (29.9) | 40 (19.0) |
| **Cephalosporins** | Cefepime | 467 | 372 (79.7) | 55 (11.8) | 40 (8.6) |
|  | Ceftazidime | 469 | 366 (78.0) | 73 (15.6) | 30 (6.3) |
|  | Ceftazidime-avibactam | 28 | 18 (64.3) | 10 (35.7) | 0 (0.0) |
|  | Ceftolozane-tazobactam | 6 | 6 (100.0) | 0 (0.0) | 0 (0.0) |
| **Carbapenems** | Imipenem | 463 | 326 (70.4) | 113 (24.4) | 24 (5.2) |
|  | Meropenem | 353 | 240 (68.0) | 97 (27.5) | 16 (4.5) |
| **Monobactam** | Aztreonam | 132 | 94 (71.2) | 30 (22.7) | 8 (6.1) |
| **Aminoglycosides** | Amikacin | 467 | 420 (89.9) | 43 (9.0) | 4 (0.9) |
|  | Gentamicin | 447 | 390 (87.2) | 34 (7.6) | 23 (5.1) |
|  | Tobramycin | 332 | 305 (91.6) | 26 (7.8) | 2 (0.6) |
| **Quinolones** | Ciprofloxacin | 471 | 340 (71.3) | 96 (20.4) | 35 (7.4) |
|  | Levofloxacin | 34 | 20 (58.8) | 12 (35.3) | 2 (5.9) |
|  | Pefloxacin | 203 | 130 (64.0) | 53 (26.1) | 20 (9.9) |
| **Polymyxins** | Colistin | 447 | 443 (99.1) | 2 (0.4) | 2 (0.4) |

^a^ Number of isolates tested with each antibiotic. ‎
