# Supplementary material for: Difficult-to-treat resistant Pseudomonas aeruginosa infections in Lebanese hospitals: Impact on mortality and the role of initial antibiotic therapy
Source: PLoS One. 2025 May 12;20(5):e0321935. doi: 10.1371/journal.pone.0321935 (PMC12068612; doi:10.1371/journal.pone.0321935)
Supplement: Attachment — Submitted filename: Response to Reviewers.docx [file pone.0321935.s003.docx]

**Dear Prof. Giuseppe Pipitone, MD**
Academic Editor
PLOS ONE

PONE-D-24-46888

Thank you for giving us the opportunity to submit a revised draft of our manuscript titled “**Difficult-to-Treat Resistant *Pseudomonas aeruginosa* Infections in Lebanese Hospitals: Impact on Mortality and the Role of Initial Antibiotic Therapy**” to PLOS ONE. We appreciate the time ‎and effort that you and the reviewer have dedicated to providing valuable feedback on our ‎manuscript. We are grateful to your insightful comments. We have been able to ‎incorporate changes to reflect all of the suggestions you and the reviewer have provided. Those changes are ‎highlighted within the manuscript. ‎

Please see below, in **blue**, for point-by-point responses to the comments and ‎concerns.

**Reviewers' comments:**

Reviewer's Responses to Questions

**Comments to the Author**

1. Is the manuscript technically sound, and do the data support the conclusions?

The manuscript must describe a technically sound piece of scientific research with data that supports the conclusions. Experiments must have been conducted rigorously, with appropriate controls, replication, and sample sizes. The conclusions must be drawn appropriately based on the data presented.

Reviewer #1: Partly

**Authors’ response:**

Dear Reviewer. Thank you for taking the time to extensively review our manuscript and for providing valuable comments and suggestions. We sincerely appreciate your insightful feedback, which has significantly contributed to improving the quality and clarity of our work. We have thoroughly reviewed each of your comments and made the appropriate revisions. Below, you will find our detailed responses to your suggestions, along with the changes implemented in the manuscript. We hope these revisions meet your expectations and that the manuscript is now suitable for publication.

2. Has the statistical analysis been performed appropriately and rigorously?

Reviewer #1: Yes

3. Have the authors made all data underlying the findings in their manuscript fully available?

The [PLOS Data policy](https://eur01.safelinks.protection.outlook.com/?url=http%3A%2F%2Ftrack.editorialmanager.com%2FCL0%2Fhttp%3A%252F%252Fwww.plosone.org%252Fstatic%252Fpolicies.action%2523sharing%2F1%2F010f01940762ca77-e74db438-7e1e-4d63-9582-b91c07061862-000000%2F99vq5wn2DS9mifm8BsOEDkXCb4ifAO5vyVtnp5V8ERc%3D190&data=05%7C02%7Cr.itani%40bau.edu.lb%7C0c5827a011fc49ba6b3408dd26567c04%7Cae73320887834b9394a5c5f29fb71fa9%7C0%7C0%7C638708875551771112%7CUnknown%7CTWFpbGZsb3d8eyJFbXB0eU1hcGkiOnRydWUsIlYiOiIwLjAuMDAwMCIsIlAiOiJXaW4zMiIsIkFOIjoiTWFpbCIsIldUIjoyfQ%3D%3D%7C0%7C%7C%7C&sdata=6B0MJhiAUKFqpvQTY47ifcr2W3BATu5doQafMaQgxPE%3D&reserved=0) requires authors to make all data underlying the findings described in their manuscript fully available without restriction, with rare exception (please refer to the Data Availability Statement in the manuscript PDF file). The data should be provided as part of the manuscript or its supporting information, or deposited to a public repository. For example, in addition to summary statistics, the data points behind means, medians and variance measures should be available. If there are restrictions on publicly sharing data—e.g. participant privacy or use of data from a third party—those must be specified.

Reviewer #1: No

**Authors’ response:**

We fully understand the importance of making underlying data available. However, the datasets generated and analyzed during this study cannot be publicly available due to restrictions imposed by the Institutional Review Boards (IRBs) and ethical committees of the participating hospitals. These restrictions are in place to protect patient privacy and confidentiality, as the data contain potentially identifying or sensitive patient information. Public sharing of this data could risk patient re-identification. Additionally, given that this study assessed hospital management practices, further confidentiality concerns apply. In accordance with ethical standards, access to these datasets is possible only upon formal request to the corresponding author (r.itani@bau.edu.lb) or to the IRB at Beirut Arab University (irb@bau.edu.lb), accompanied by approval from the hospitals' IRBs and ethical committees. All relevant data supporting the conclusions of this study have been included within the article. Furthermore, the data will be securely stored in Beirut Arab University’s institutional repository, ensuring long-term availability and accessibility through the IRB. We have incorporated these details into the **Data Availability Statement** in the manuscript.

4. Is the manuscript presented in an intelligible fashion and written in standard English?

PLOS ONE does not copyedit accepted manuscripts, so the language in submitted articles must be clear, correct, and unambiguous. Any typographical or grammatical errors should be corrected at revision, so please note any specific errors here.

Reviewer #1: Yes

5. Review Comments to the Author

Please use the space provided to explain your answers to the questions above. You may also include additional comments for the author, including concerns about dual publication, research ethics, or publication ethics. (Please upload your review as an attachment if it exceeds 20,000 characters)

**Reviewer #1:** The authors submitted a manuscript investigating Pseudomonas aeruginosa infections in 4 Lebanese tertiary care hospitals, with a particular focus on the prevalence and impact of DTR isolates, as well as the appropriateness of initial antibiotic therapy and predictors of 30-day in-hospital mortality.

- The manuscript describes exclusion criteria (e.g., <48 h hospital stay, incomplete records, co-infections). This is raises the risk of selection bias. please expand on the rationale for excluding patients discharged or leaving the hospital within 48 hours (this will be further touched upon later in my report). Were some of those patients potentially stable, or were they left out simply because they might not have had time to develop or treat an infection? The flow diagram (Figure 1) is helpful, but more detail on potential biases from these exclusions would be beneficial.

**Authors’ response**:

Thank you for your thoughtful comment and for highlighting the potential risk of selection bias introduced by the exclusion criteria. We appreciate the opportunity to clarify the rationale for excluding patients discharged or leaving the hospital within 48 hours, as well as the potential biases associated with this decision. Patients discharged or who left the hospital within 48 hours of infection acquisition were excluded if they did not receive their initial antibiotic therapy within the hospital. Since our study focuses on assessing the appropriateness of initial antibiotic therapy administered during hospitalization, including these patients would have limited our ability to evaluate this aspect consistently. Additionally, discharge medications were not systematically documented in this study, further limiting our ability to assess post-discharge antibiotic use and its influence on treatment outcomes. We have handled these issues as follows:

**2. Methods Section**

***2.2. Study population***

“The study included hospitalized adult patients aged 18 years and older with a confirmed diagnosis of *P. aeruginosa* infection who received treatment at any of the participating hospitals during the study period. For patients with recurrent *P. aeruginosa* infections, only the first infection episode was documented to avoid overrepresentation of individual patients in the dataset and to reflect baseline antimicrobial resistance profiles without the confounding influence of prior treatments or recurrent infections.

The exclusion criteria were as follows: cases of colonization rather than active infection, polymicrobial infections or co-infections, patients treated as outpatients (i.e., those not admitted to the hospital), subsequent hospitalizations due to recurrent infections, patients discharged or who left the hospital before receiving initial antibiotic therapy, and incomplete medical records lacking antimicrobial susceptibility testing (AST) results or other crucial medical information. These criteria were designed to establish a focused and representative study population of hospitalized patients with active *P. aeruginosa* infections. By excluding cases involving recurrent infections, co-infections, or colonization, the study aimed to minimize confounding factors that could obscure the specific impact of *P. aeruginosa* on clinical outcomes. This approach enabled a targeted evaluation of the appropriateness of initial antibiotic therapy for infections caused by *P. aeruginosa* and facilitated the estimation of the associated in-hospital mortality.”

***Study limitations*** (3^rd^ paragraph)

“Third, by focusing solely on hospitalized patients who received their initial antibiotic therapy within the hospital, the study excluded outpatients, potentially leading to an overestimation of resistance patterns. As a result, the observed trends likely reflected more severe infections requiring hospitalization, limiting the applicability of the findings to less severe cases managed in outpatient settings. Fourth, the decision to include only the first infection episode for each patient excluded follow-up infections and cultures, which might have provided valuable insights into disease progression, treatment response, and the evolution of antimicrobial resistance. This approach may have introduced a bias toward simpler cases, potentially overlooking the complexities of recurrent or prolonged infections. Fifth, the exclusion of polymicrobial infections, which are frequently encountered in clinical practice, limited the study's applicability to real-world scenarios. Polymicrobial infections often involve interactions between multiple pathogens, affecting disease severity, resistance patterns, and treatment outcomes. Excluding these cases may have overlooked important challenges in managing such infections.”

We also revised **Figure 1** to provide additional detail on the exclusion of patients discharged without receiving initial antibiotic therapy during their hospital stay.

- This endpoint can overestimate infection-attributable deaths. The authors acknowledge this limitation. discuss how infection-related mortality was confirmed, or whether chart reviews attempted to differentiate conflicting causes of death.

**Authors’ Response:**

Thank you for your insightful comment. We agree that the use of 30-day all-cause mortality as the primary endpoint may overestimate infection-attributable deaths, as not all deaths within this timeframe can be linked to *P. aeruginosa* infection. This limitation is inherent to our study's retrospective design and the reliance on medical records, which often lack explicit documentation of the exact cause of death.

In our chart reviews, we aimed to identify potential contributors to mortality; however, detailed clinician-assigned cause-of-death information was not consistently available. This limitation restricted our ability to differentiate infection-related deaths from those caused by underlying comorbidities, other infections, or non-infectious conditions. We have acknowledged this challenge in the ***Study limitations*** section (4^th^ paragraph), as follows:

“Furthermore, the use of 30-day all-cause mortality as the primary endpoint likely led to an overestimation of infection-attributable deaths, as not all deaths within this timeframe could be conclusively attributed to *P. aeruginosa* infection. The retrospective design and the lack of explicit documentation in medical records further limited our ability to determine the exact causes of death. Consequently, the results should be interpreted with caution, as they may overstate the mortality burden directly associated with *P. aeruginosa* infections.”

a. A Kaplan-Meier with log-rank comparisons for time-to-mortality is used. The difference for time-to-event between DTR vs. non-DTR just misses significance (p=0.07), but the difference based on appropriateness of initial therapy is significant (p=0.03). The text reports “mean survival time” and 95% CIs that appear somewhat narrow (e.g., for the DTR group in Table 4: 4.06–7.93 days?). The survival curves appear to go out to 30 days; thus, the meaning of that confidence interval needs more clarity. Are these the 95% CIs for median survival times?

**Authors’ response:**

Thank you for your valuable observation. You are correct that the 95% confidence intervals (CIs) reported for survival times in **Table 3** and **Table 4** were originally for the **median** survival time rather than the **mean**. We acknowledge that this distinction could lead to confusion for readers, especially since Kaplan-Meier curves typically extend to 30 days while the reported CIs for the DTR group were relatively narrow (e.g., 4.06–7.93 days). To address this, we have revised **Table 3** and **Table 4** to now report the mean survival time and its 95% CI, as this provides a more straightforward and interpretable metric in this context.

b. The approach to univariate → multivariable logistic regression is described, with a p < 0.2 cut-off for inclusion. The text says that forward stepwise selection was used, and that variance inflation factors (VIF > 10) triggered exclusion. This is reasonable, but it is unclear which variables were excluded due to collinearity, I will mention this again below in detailed analysis. More detail would help: for instance, was the site of infection acquisition (ICU vs. non-ICU) collinear with nosocomial vs. community infection? Also, the final model includes female sex, DTR infection, and appropriateness of therapy. Yet the univariate table also identified age ≥ 65, CCI, and glucocorticoid use as having p<0.2. Possibly these were excluded for collinearity reasons, but clarifying this in the text would be important to ensure transparency.

**Authors’ response:**

Thank you for your valuable comment and for highlighting the need for additional clarity regarding variable inclusion and exclusion in the multivariable logistic regression model. We have revised the methodology section as follows:

***2.6. Statistical analysis*** (2^nd^, 3^rd^, and 4^th^ paragraphs)

“Univariate analysis was conducted to assess associations between independent variables and 30-day all-cause mortality, utilizing Pearson's chi-square (χ²) test for categorical variables and independent t-tests for continuous variables. Variables with a P-value < 0.2 in the univariate analysis were included in the multivariable logistic regression model. The variables initially included were: age group (≥ 65 years vs. 18–64 years), sex (female vs. male), site of infection acquisition (community-acquired vs. nosocomial infection), hospital department of infection acquisition (ICU vs. non-ICU), coronary artery disease, congestive heart failure, CCI, previous glucocorticoid use within the last four weeks, type of antimicrobial resistance (DTR vs. non-DTR *P. aeruginosa* infection), and appropriateness of the initial antibiotic therapy (appropriate vs. inappropriate).

Multicollinearity was assessed using variance inflation factors (VIF), with a threshold of 10 for exclusion. All VIF values were below 2.5, indicating no significant multicollinearity among the predictors. Conceptually related variables were carefully reviewed for inclusion based on clinical relevance and contribution to model fit. After careful consideration, ICU vs. non-ICU was retained for its stronger relevance to patient outcomes, while nosocomial vs. community-acquired infection was excluded. Similarly, congestive heart failure and coronary artery disease were excluded due to their conceptual overlap with CCI, which was retained as a more comprehensive measure of comorbidity burden.

The final multivariable logistic regression model was developed using a stepwise forward selection approach, which sequentially added variables based on their statistical contribution to the model. Confounding variables were retained in the model if their inclusion altered the coefficient of any significant variable by 10% or more. The glucocorticoid use, although identified in the univariate analysis (P < 0.2), was excluded during forward selection as it did not significantly contribute to the final model after accounting for other variables. The goodness-of-fit for the model was assessed using Hosmer-Lemeshow test. Adjusted odds ratios (AOR) with 95% confidence intervals and P-values were calculated for all variables in the model.”

c. The mortality rate of 14.8% fits within expected estimates. The discussion section offers does not offer sufficient rationale or thoughts as to why female patients had higher mortality. Assuming that any difference in comorbidity that is gender based would be accounted for in a logistic regression model; please expand on potential epidemiologic reasons (e.g., healthcare-access?). Again this will be mentioned in more detail below

**Authors’ response:**

Thank you for your valuable feedback and for highlighting this important issue. We have considered your suggestion **mentioned below** and conducted additional analyses to assess the potential confounding effect of sex on mortality. Specifically, we examined the distribution of comorbidities, Charlson Comorbidity Index (CCI), and ICU admission by sex. Chi-square tests were performed for categorical variables, and independent t-tests were used for continuous variables (CCI). This analysis revealed significant differences in the distribution of these factors between male and female patients, suggesting that they could influence the observed mortality rates. In light of these findings, we refined our multivariable logistic regression model to account for comorbidity burden (CCI) and ICU admission as potential confounders. After incorporating these adjustments, the previously observed association between female sex and mortality was no longer statistically significant. This has been reflected in the revised manuscript.

d. Despite the non-significant p=0.07 in the survival analysis, the logistic regression finds DTR to be an independent predictor of mortality (AOR 3.11). This discrepancy likely arises from how survival times were partitioned and the additional effect of confounders in the multivariable model. Please provide provide a short note about this distinction in the Discussion. Some reasons may require interpretation as to why the log-rank p-value for time-to-mortality differs from the logistic regression results.

**Authors’ response:**

Thank you for highlighting this important issue. We have addressed this discrepancy in the **Discussion** Section (17^th^ paragraph) as per your suggestion as follows:

“The Kaplan-Meier survival analysis with the log-rank test did not detect a statistically significant difference in survival times between patients with DTR and non-DTR *P. aeruginosa* infections (P = 0.07). However, logistic regression identified DTR infections as a significant independent predictor of 30-day mortality (AOR = 2.48, P < 0.001). This discrepancy likely arises from fundamental differences in analytical approaches. Kaplan-Meier analysis evaluates unadjusted time-to-event distributions and does not account for confounding variables or potential violations of proportional hazards assumption. In contrast, logistic regression examines the overall association between DTR infections and mortality while adjusting for confounders, thereby isolating the independent effect of DTR infections on mortality. While Kaplan-Meier analysis focuses on survival time distributions and incorporates censored data, logistic regression simplifies the outcome into a binary variable (survival vs. death), providing a clear focus on overall mortality risk (61–63). Thus, even if survival time distributions between DTR and non-DTR groups do not differ significantly, the logistic regression analysis demonstrates a significantly higher overall mortality risk in the DTR group. These methodological differences emphasize the importance of employing complementary statistical methods to comprehensively assess the impact of DTR *P. aeruginosa* infections on mortality outcomes.”

e. More detail on how the authors handled or imputed missing data (if any was imputed at all) is needed.

**Authors’ response:**

Thank you for highlighting the importance of addressing missing data. We confirm that this aspect was addressed in the study methodology. Patients’ files were thoroughly screened for completeness, and a total of 87 cases were excluded due to missing data or incomplete medical records. These exclusions primarily involved files lacking antimicrobial susceptibility testing (AST) results or other critical medical information necessary for the analysis. We acknowledge that excluding these cases may have introduced a potential selection bias. However, this approach was deemed necessary to ensure the accuracy and reliability of the dataset used in the study. This clarification has been emphasized in the revised Methods section of the manuscript.

f. As stated a major data gap is post-discharge therapy and outcomes which is understandable given the study’s retrospective design, but the authors might mention whether they attempted to collect data from outside the hospital within that context for some patients or if that was uniformly unavailable.

**Authors’ Response:**

Thank you for pointing this out. We acknowledge that the lack of data on post-discharge therapy and outcomes represents a notable gap in the study. Given the retrospective nature of the study, our data collection was limited to hospital records, which did not routinely include follow-up information after discharge. We did not attempt to collect data from outpatient clinics, external sources, or directly contact the patients post-discharge, as this information was uniformly unavailable for all patients included in the study.

We agree that post-discharge data would have provided valuable insights into the long-term impact of antimicrobial therapy and infection recurrence. This limitation has now been explicitly acknowledged and discussed in the ***Study limitations*** section (5^th^ paragraph) as follows:

“Additionally, the study only documented definitive antibiotic therapy administered during the hospital stay and did not account for therapy or outcomes following patient discharge. Due to the retrospective design, post-discharge data, including outpatient therapy and long-term outcomes, were systematically collected and were uniformly unavailable for all patients. This omission limits our ability to evaluate the influence of post-discharge antibiotic therapy on infection recovery, recurrence, and overall patient outcomes.”

- Did all four hospitals use the same AST platforms and interpretative standards (CLSI vs. EUCAST)?

**Authors’ response:**

We thank you for this valuable comment. We have now detailed in the Methods section the microbiological identification, AST platforms used, and the interpretative standards followed by the participating hospitals, as follows:

*“****2.3. Microbiological identification and antimicrobial susceptibility testing*** (1^st^, 2^nd^, and 3^rd^ paragraphs)

Microbiological identification and AST of *P. aeruginosa* were conducted at four participating hospitals, each following its respective laboratory protocols. In Hospitals 1 and 2, bacterial identification and AST were performed using the VITEK 2 automated system (bioMérieux, Marcy-l'Étoile, France), which determines the minimum inhibitory concentrations (MICs) of tested antibiotics. In contrast, Hospitals 3 and 4 employed the Kirby-Bauer disk diffusion method, following the Clinical and Laboratory Standards Institute (CLSI) guidelines. Inhibition zone diameters were measured manually and verified using the ADAGIO automated zone reader (Bio-Rad Laboratories, Hercules, CA, USA).

AST results for all hospitals were interpreted according to CLSI-defined breakpoint criteria (19). The susceptibility of *P. aeruginosa* isolates was classified as susceptible (S), intermediate (I), or resistant (R).

Routine AST included a panel of β-lactams (piperacillin-tazobactam, ceftazidime, cefepime, meropenem or imipenem, and aztreonam), aminoglycosides (gentamicin, tobramycin, and amikacin), fluoroquinolones (ciprofloxacin), and polymyxins (colistin). In cases where resistance to standard β-lactams was detected, additional AST was performed in one hospital against novel β-lactam/β-lactamase inhibitor combinations, including ceftazidime-avibactam and ceftolozane-tazobactam, to assess their potential efficacy against resistant strains.”

- Table 1 indicates that 6 patients received CAZ-AVI empirically. Please provide context for this empiric practice and whether the AST associated with these 6 patients justified appropriate CAZ-AVI use.

**Authors’ response:**

Thank you for highlighting this important aspect. Six patients in one of the participating hospitals received ceftazidime-avibactam (CAZ-AVI) empirically. CAZ-AVI was initiated in these patients due to their high-risk clinical conditions, which included infections acquired in the intensive care unit following prolonged hospital stays, urosepsis, complicated intra-abdominal infections, and ventilator-associated pneumonia. Additionally, two of these patients had underlying chronic kidney disease, and one had previously received carbapenem therapy for a different infection.

The appropriateness of this empiric therapy was assessed based on predefined criteria for empirical antibiotic selection. Specifically, “The appropriateness of the initial antibiotic therapy was assessed by determining whether one or more antibiotic agents were administered within 48 hours of infection onset, and whether the causative pathogen was susceptible to these agents according to AST results. The antibiotics must have been delivered via the correct route and at an appropriate dosage regimen. Any initial antibiotic therapy that did not meet these criteria was considered inappropriate.” Based on these criteria, all six patients received appropriate empirical therapy, with the causative pathogens being susceptible to CAZ-AVI. Four of the patients were infected with DTR *P. aeruginosa*, while two had non-DTR *P. aeruginosa* and were also found to be susceptible to CAZ-AVI.

Regarding definitive therapy, four patients continued on CAZ-AVI appropriately, while one patient was inappropriately continued on CAZ-AVI despite the availability of narrower-spectrum alternatives. The remaining patient was de-escalated to oral ciprofloxacin following susceptibility confirmation. It is important to note that empiric CAZ-AVI use is not a routine practice in Lebanese hospitals, and its administration was based on clinical judgment, supported by the severity of the patients’ conditions. We have detailed this in the study **Discussion** (10^th^ paragraph) as follows:

“The empirical use of ceftazidime-avibactam (CAZ-AVI) was observed in six patients at one of the participating hospitals, driven by high-risk clinical conditions, including ICU-acquired infections following prolonged hospital stays, urosepsis, complicated intra-abdominal infections, and ventilator-associated pneumonia. Two patients had underlying chronic kidney disease, and one had prior carbapenem therapy. While empiric CAZ-AVI use is not a routine in Lebanese hospitals, its administration was based on clinical judgment, considering infection severity and MDR risk factors. Although the use of empirical CAZ-AVI is not recommended for the treatment of antimicrobial resistant Gram-negative infections, according to the guidelines of the Infectious Diseases Society of America (IDSA), its justification in high-risk patients with suspected MDR Gram-negative infections is acknowledged (50). Empiric selection should be guided by local resistance patterns, patient-specific factors, prior antibiotic exposure, infection source, and illness severity. Thus, CAZ-AVI should be reserved for targeted therapy, and its empirical use should be restricted through strengthening AMS practices to optimize empirical antibiotic selection and minimize resistance development.”

-The same can be said about the empiric usage of several agents such as ceftaroline, linezolid, and colistin. Please elaborate on whether these are errors. By empiric, most readers will consider this to represent Pre-AST.

**Authors’ Response:**

Thank you for your comment. We have addressed this point in the **Discussion** section (11^th^ and 12^th^ paragraphs) as follows:

“This study documented instances where broad-spectrum last-line antibiotics were used empirically before obtaining AST results. These antibiotics, including colistin, linezolid, and ceftaroline, are typically reserved for salvage therapy rather than routine empirical use. Upon reviewing the cases in which these antibiotics were initiated empirically, most patients presented with critical illnesses and severe infections, such as ventilator-associated pneumonia (VAP), bloodstream infections, complicated skin and soft tissue infections (cSSTIs), and infections acquired in ICU settings. Additionally, many patients developed sepsis or septic shock. While the literature strongly discourages the empirical use of last-resort antibiotics and emphasizes their role in targeted therapy, their administration may be justified in selected high-risk cases. For instance, empirical colistin therapy may be warranted in critically ill patients with invasive infections and a high likelihood of infection with XDR pathogens. Specific scenarios where empirical colistin use may be appropriate include ICU patients with VAP in settings with a high prevalence of MDR *Acinetobacter baumannii* or *P. aeruginosa*, as well as severe nosocomial infections in regions with high rates of carbapenem resistance. However, due to colistin’s significant nephrotoxicity, its use should be restricted to situations where no alternative options exist (50–53). Similarly, the IDSA recommends empirical linezolid use in cases of hospital-acquired pneumonia (HAP) or VAP, cSSTIs, or bacteremia when methicillin-resistant *Staphylococcus aureus* (MRSA) is strongly suspected, especially in patients with risk factors such as nasal colonization, prior MRSA infection, recent hospitalization, or recent antibiotic exposure (54–56).

In Lebanon, the increasing prevalence of MDR pathogens—including carbapenem-resistant *A. baumannii* (CRAB), carbapenem-resistant *Klebsiella pneumoniae* (CRKP), carbapenem-resistant *P. aeruginosa*, and MRSA—has led to a growing use of last-line antibiotics, as these pathogens are often resistant to all other available treatment options (20,25,57). However, the overuse of these critical antibiotics poses a serious threat to AMS efforts and may contribute to further resistance development. Given these concerns, future research should assess the appropriateness of prescribing patterns for last-line antibiotics and establish clear criteria for their use. A risk-based approach should be defined to identify specific conditions and patient profiles that warrant their initiation. Additionally, restricting access to these antibiotics—requiring approval from a multidisciplinary team comprising infectious disease specialists, clinical microbiologists, and clinical pharmacists—would be a crucial step toward ensuring their judicious use and preserving their efficacy for truly resistant infections.”

Given that data from your region is harder to come by your cohort can reveal more and inform more about the epidemiology:

- Please inform readers of the routine AST agents performed for pseudomonas aeruginosa. Is CAZ-AVI susceptibility testing reflex tested with carbapenem non-susceptibility?

**Authors’ response:**

Thank you for your valuable comment. We have now specified the routine AST agents tested for Pseudomonas aeruginosa in the participating hospitals under the methodology section. Regarding CAZ-AVI susceptibility testing, it was not performed routinely across all hospitals. However, in one of the participating hospitals, CAZ-AVI testing was conducted when resistance to standard β-lactams was detected, as outlined below:

“***2.3. Microbiological identification and antimicrobial susceptibility testing*** (2^nd^ and 3^rd^ paragraphs)

AST results for all hospitals were interpreted according to CLSI-defined breakpoint criteria (19). The susceptibility of P. aeruginosa isolates was classified as susceptible (S), intermediate (I), or resistant (R).

Routine AST included a panel of β-lactams (piperacillin-tazobactam, ceftazidime, cefepime, meropenem or imipenem, and aztreonam), aminoglycosides (gentamicin, tobramycin, and amikacin), fluoroquinolones (ciprofloxacin), and polymyxins (colistin). In cases where resistance to standard β-lactams was detected, additional AST was performed in one hospital against novel β-lactam/β-lactamase inhibitor combinations, including ceftazidime-avibactam and ceftolozane-tazobactam, to assess their potential efficacy against resistant strains.”

- What percentage of isolates were resistant to CAZ-AVI?

**Authors’ response:**

Thank you for your comment. Testing for ceftazidime-avibactam (CAZ-AVI) was performed on only 28 isolates from one of the participating hospitals. We have now clarified this within the 2^nd^ paragraph of **Section 3.3** under the **Results**, as follows:

“Out of the 28 isolates tested for ceftazidime-avibactam, 10 isolates (10/28, 35.7%) were resistant.”

- Is Ceftolozane-tazobactam available in any of the medical centers included in the study? If yes, what % os isolates were non-susceptible to TOL-TAZ?

**Authors’ response:**

Thank you for your inquiry. Yes, ceftolozane-tazobactam (TOL-TAZ) is available in the participating hospitals, although its use is still limited. A total of six isolates were tested against ceftolozane-tazobactam, and all were found to be susceptible. We have now clarified this within the 2^nd^ paragraph of **Section 3.3** under the **Results**, as follows:

“In contrast, the majority of isolates remained susceptible to tobramycin (91.6%), amikacin (89.9%), and gentamicin (87.2%). Out of the 28 isolates tested for ceftazidime-avibactam, 10 isolates (10/28, 35.7%) were resistant. Meanwhile, all six isolates tested for ceftolozane-tazobactam (6/6, 100%) were susceptible **(*Supplementary Appendix, Table S1*)**.”

- Is Cefiderocol available in any of the medical centers included in the study? Was resistance encountered?

**Authors’ response:**

Thank you for your inquiry. Cefiderocol is not currently available in the Lebanese market, and it was not tested in any of the participating hospitals. Consequently, resistance to cefiderocol was not encountered in our study.

- Did you encounter on follow up cultures resistance to any B-lactam derived agents including cephalosporins or carbapenems or CAZ/AVI during treatment

**Authors’ response:**

Thank you for your comment. We acknowledge that follow-up cultures were not included in our study, as we focused solely on the first culture obtained from each patient. The study did not document subsequent cultures, and therefore, resistance patterns observed during treatment or after follow-up were not assessed. We have included this limitation in the study under ***Study limitations*** (within 2^nd^ paragraph and within 3^rd^ paragraph), as follows:

“Second, the retrospective design and reliance on medical records for data collection present inherent limitations. Critical information that may not have been documented could have been omitted, potentially affecting the completeness of the analysis. The absence of key data restricts our ability to establish causal relationships, and the inability to account for certain confounding variables may hinder our capacity to accurately identify mortality predictors associated with DTR *P. aeruginosa* infections. For example, essential variables such as admitting diagnoses, laboratory results, follow-up cultures, clinical responses to treatment, and infection recurrence were not consistently retrieved from medical records. The lack of this information limits the depth of our analysis and introduces potential bias when evaluating factors that influence patient outcomes. Furthermore, although we accounted for comorbidities using the CCI, we were unable to include baseline severity scores (e.g., APACHE II, SOFA) due to incomplete documentation. These scores could have provided additional insights into the impact of disease severity on patient outcomes. The absence of follow-up cultures specifically restricted our ability to assess how resistance patterns may have evolved during treatment.”

“Fourth, the decision to include only the first infection episode for each patient excluded follow-up infections and cultures, which might have provided valuable insights into disease progression, treatment response, and the evolution of antimicrobial resistance. This approach may have introduced a bias toward simpler cases, potentially overlooking the complexities of recurrent or prolonged infections.”

-	For definitive antibiotic therapy, also seen in Table 2; I am assuming Fosfomycin is the IV formulation. Please indicate (IV) in brackets in the table. Many readers are only familiar with PO FOS in the setting of UTIs.

**Authors’ response:**

Thank you for your valuable comment. In Lebanon, only the oral formulation of Fosfomycin (granules for solution) is available, and the intravenous (IV) formulation is not present in the market. The four patients who received Fosfomycin as definitive antibiotic therapy were treated with the oral formulation (PO). We have updated **Table 2** to reflect this information.

- You mention the absence of national surveillance data and “the paucity of well-designed studies” on DTR PA in Lebanon. Are there any recent steps taken nationally to track AMR? A few lines referencing the challenges of implementing surveillance is helpful in epidemiologic studies.

**Authors’ response:**

Thank you for your insightful comment. We have now incorporated details on recent national efforts to track AMR and the challenges of implementing national surveillance in the **Background** section (3^rd^, 4^th^, And 5^th^ paragraphs), as follows:

**“Despite growing concern over DTR *P. aeruginosa* due to its high risk of treatment failure and significant impact on patient outcomes, there is a paucity of well-designed studies investigating the impact of DTR *P. aeruginosa* infections on mortality. This gap is particularly pronounced in Lebanon, a lower-middle-income country with a fragile healthcare infrastructure, which lacks national surveillance data and robust epidemiological tracking. Lebanon’s challenges in controlling AMR arise from underdeveloped informatics systems, weak health systems governance, limited resources, poorly implemented antimicrobial stewardship (AMS) programs, and an absence of governmental oversight regarding antibiotic dispensing practices** (10–13)**.**

Lebanon has undertaken several initiatives at the national level to address AMR. In alignment with global surveillance efforts, the country joined the World Health Organization’s (WHO) Global Antimicrobial Resistance Surveillance System (GLASS) to facilitate AMR data reporting and integrate laboratory data into international systems (14,15). However, Lebanon has yet to submit AMR data to GLASS, as it is still in the process of building a national AMR surveillance system (16). In 2019, the Ministry of Public Health (MoPH) established a National Antibiotic Resistance Committee and an AMR Task Force, which drafted a National Action Plan (NAP) to enhance AMR surveillance. The plan aimed to map laboratories for microbiologically reliable data, issue periodic national AMR reports, promote rational antimicrobial use, establish AMS programs in hospitals, develop national treatment guidelines for infectious diseases, and strengthen infection prevention and control measures (17).

Despite these efforts, the AMR-NAP was not officially integrated into the national health plan due to ongoing political instability, economic crises, and deficiencies in essential infrastructure, such as electricity and waste management. The lack of dedicated funding and financial investment further hindered its implementation. Consequently, national AMR surveillance data remain scarce, primarily confined to retrospective, single-center, or multi-center studies conducted in tertiary care settings. Available data are fragmented and lack epidemiological representativeness, limiting their utility in guiding policymakers and effectively allocating resources to mitigate antimicrobial resistance (16,18).”

- With regards to the 4 hospitals in Beirut city, do they differ in patient populations, bed capacity, or infection-control measures?

**Authors’ response:**

Thank you for your insightful comment. In response to your recommendation, we have provided a detailed description of the differences among the participating hospitals regarding patient population, bed capacity, and infection prevention and control measures. This information has been incorporated into the **Methodology** section as follows:

*“****2.1. Study design, setting, and institutional characteristics***

This retrospective, multicenter study was conducted across four large teaching hospitals in Beirut, Lebanon. It included patients hospitalized with *P. aeruginosa* infections over a three-year period, from January 1, 2021, to December 31, 2023. Data were accessed for research purposes, and data collection began on February 1, 2024.

The participating hospitals comprise three private, university-affiliated hospitals and one public teaching hospital. The private hospitals provide medical services to individuals from diverse socioeconomic backgrounds, while the public hospital primarily serves low-to-middle-income patients. These institutions offer specialized healthcare services, collectively addressing a broad spectrum of medical needs within the Lebanese community. The bed capacities vary, with approximately 75 beds in one hospital, 97 beds in another, and 250 beds each in the third and fourth hospitals.

All participating hospitals have Infection Prevention and Control (IPC) committees and, to varying extents, AMS programs. However, the structure, implementation, AMR containment efforts, and clinician compliance differ among the participating institutions. The private hospitals have well-established AMS and IPC committees, consisting of infectious disease specialists, clinical pharmacists, microbiologists, infection control officers, laboratory technicians, information technology specialists, and hospital epidemiologists. These committees oversee antibiotic prescribing practices, infection control measures, compliance with standard and transmission-based precautions, and proper sterilization procedures for hospital units and medical equipment. Additionally, they facilitate patient and visitor education, conduct epidemiological studies, and provide training sessions for healthcare professionals.

In contrast, the public hospital has an IPC committee composed of infectious disease specialists and nursing staff but lacks a formal AMS committee. This results in the absence of structured AMS protocols, antimicrobial susceptibility surveillance reports, and the enforcement of antimicrobial prescription through preauthorization policies.”

- Were patients <48 hrs excluded because they might not have had time to be fully evaluated or treated? Other reasons? Please quantify the effect of excluding from your study patients who left the hospital within 48 hours of acquiring infection. How does this exclusion impact the observed results and conclusions. A bias may have been inadvertently introduced by excluding mild infections that had resolved more rapidly. This is relevant to the observed significant findings. How might this exclusion have impacted the stepwise model, and ultimately across significant subgroups as it pertains to primary outcomes.

**Authors’ response:**

Thank you for your inquiry. Patients who were discharged or left the hospital within 48 hours of infection acquisition were excluded if they did not receive their initial antibiotic therapy within the hospital. Since our study aims to evaluate the appropriateness of initial antibiotic therapy administered during hospitalization, including these patients would have introduced inconsistencies in our assessment. Exclusion was not based solely on the duration of hospitalization but rather on the administration of the initial antibiotic therapy. To enhance clarity, we have revised the **Methods section** accordingly to ensure there is no ambiguity regarding the exclusion criteria. We have also emphasized the potential biases in the ***Study limitations*** (3^rd^ paragraph) as follows:

“Third, by focusing solely on hospitalized patients who received their initial antibiotic therapy within the hospital, the study excluded outpatients, potentially leading to an overestimation of resistance patterns. As a result, the observed trends likely reflected more severe infections requiring hospitalization, limiting the applicability of the findings to less severe cases managed in outpatient settings. Fourth, the decision to include only the first infection episode for each patient excluded follow-up infections and cultures, which might have provided valuable insights into disease progression, treatment response, and the evolution of antimicrobial resistance. This approach may have introduced a bias toward simpler cases, potentially overlooking the complexities of recurrent or prolonged infections. Fifth, the exclusion of polymicrobial infections, which are frequently encountered in clinical practice, limited the study's applicability to real-world scenarios. Polymicrobial infections often involve interactions between multiple pathogens, affecting disease severity, resistance patterns, and treatment outcomes. Excluding these cases may have overlooked important challenges in managing such infections.”

- Did any variables meet the >10 VIF threshold and thus get excluded from the final model? If so, which ones?

**Authors’ response:**

Thank you for your inquiry. No variables met the >10 VIF threshold for exclusion. All variance inflation factor (VIF) values were below 2.5, indicating the absence of significant multicollinearity among the predictors. We have now clarified this in the **Statistical Analysis** section (beginning of the 3^rd^ paragraph) of the **Methods**, as follows:

“Multicollinearity was assessed using variance inflation factors (VIF), with a threshold of 10 for exclusion. All VIF values were below 2.5, indicating no significant multicollinearity among the predictors.”

- Including a short table of final retained variables following univariate analysis would help or otherwise incorporating, bolding, or annotating with symbols may be sufficient to show the final included variable at a glance.

**Authors’ response:**

Thank you for your valuable suggestion. We have now incorporated the dagger symbol (†) to annotate each variable retained in the final model in **Table 5**. Additionally, we have clarified this annotation in the table footnote to enhance readability and ensure that the final included variables are easily identifiable at a glance.

- How might confounding variables may shift results showing survival analysis results demonstratinhg a near-significant difference in log-rank tests for DTR vs. non-DTR (p=0.07), especially given that logistic regression finds DTR to be a significant independent predictor.

**Authors’ response:**

Thank you for your insightful comment. The log-rank test in Kaplan-Meier survival analysis was a univariate test that did not account for potential confounders. In contrast, logistic regression adjusted for multiple variables, allowing it to isolate the independent effect of DTR infection on mortality. If important confounding factors were not balanced between DTR and non-DTR groups, they may have obscured the direct relationship between DTR infection and survival in the log-rank test, resulting in a near-significant p-value (p=0.07). However, once these confounders were accounted for in the logistic regression model, the significant impact of DTR on mortality became evident. This suggested that residual confounding may have influenced the unadjusted survival analysis results.

- Given the relatively advanced age and high comorbidity burden, do you have severity-of-illness scores (e.g., APACHE II, SOFA on admission)? You mention a mean SOFA of ~5.89 for those who developed sepsis, but it might be helpful to note baseline severity if available, to interpret outcomes.

**Authors’ response:**

Thank you for your insightful comment. We acknowledge the importance of assessing baseline severity of illness to better interpret patient outcomes. In our study, the SOFA score was calculated only for patients who developed sepsis, as this score is primarily used to assess organ dysfunction in septic patients. Given that not all our patients were critically ill or admitted to the ICU, we did not utilize the APACHE II score, as its application outside the ICU setting is limited and may not accurately reflect disease severity in non-critically ill patients. However, to evaluate baseline comorbidity burden and its impact on mortality, we calculated the Charlson Comorbidity Index (CCI), which is a well-established predictor of long-term mortality risk in hospitalized patients.

- Did you observe any association between specific infection sites and higher rates of DTR or carbapenem resistance?

**Authors' Response:**

Thank you for raising this important point. In response, we examined the association between the site of infection and the acquisition of DTR *P. aeruginosa*. Our analysis revealed a significant association between specific infection sites and the acquisition of DTR strains. Specifically, infections originating from the bloodstream (odds ratio [OR] = 2.93, 95% CI = 1.06–8.06, P = 0.03) and respiratory tract (OR = 2.64, 95% CI = 1.59–4.40, P < 0.01) were independently associated with an increased risk of acquiring a DTR infection. These findings are now documented in the 5^th^ paragraph under subsection **3.3** of the **Results** section of the manuscript as follows:

“A significant association was observed between specific infection sites and the acquisition of DTR *P. aeruginosa* strains. Specifically, infections originating from bloodstream (odds ratio [OR] = 2.93, 95% CI = 1.06–8.06, P = 0.03) and respiratory tract (OR = 2.64, 95% CI = 1.59–4.40, P < 0.01) were independently associated with an increased likelihood of acquiring a DTR infection.”

While these findings are of interest, we refrain from drawing conclusions or recommending empirical antibiotic therapy targeting DTR *P. aeruginosa* for patients with bloodstream or respiratory tract infections at this stage. The impact of other potential predictors and confounders needs to be assessed through a multivariate logistic regression analysis, which will allow us to better understand the true influence of the site of infection on the acquisition of DTR *P. aeruginosa*. However, this analysis is beyond the scope of our current research and may be explored in future studies.

- For patients whose antibiotics were inappropriate due to resistance, do you have data on local antibiotic guidelines that might reduce these mismatches?

**Authors’ response:**

Thank you for this valuable comment. We acknowledge the importance of local antibiotic prescribing guidelines in reducing the risk of inappropriate initial antibiotic therapy due to resistance. While the participating hospitals did not have institutional-based antibiotic prescribing guidelines at the time of the study, national guidelines developed by the Lebanese Society of Infectious Diseases and Clinical Microbiology (LSIDCM) provide recommendations for the management of various infectious diseases in Lebanon. These guidelines include:

- Management of complicated intra-abdominal infections (2018, DOI: 10.1186/s12879-019-3829-2)
- Management of COVID-19 (2020, <https://www.moph.gov.lb/userfiles/files/News/LSIDCM.pdf>)
- Management of urinary tract infections (2017, DOI: 10.12816/0042765)
- Management of community-acquired pneumonia (2014, DOI: 10.12816/0002626)
- Management of hospital-acquired pneumonia (2021, <https://doi.org/10.21203/rs.3.rs-398183/v1>)

Given the relevance of these guidelines, future studies could assess clinicians' adherence to them, which may help further elucidate the role of guideline-based therapy in reducing inappropriate antibiotic use.

- noting that 30 patients experienced >48 hours delay in therapy initiation. Was this due to diagnostic delays, or were there other logistical/administrative reasons?

**Authors’ response:**

Thank you for highlighting this point. We have now clarified the reasons for delays in the initiation of antibiotic therapy in the **Discussion** section (within the 7^th^ paragraph) as follows:

“Additionally, some patients experienced delays in receiving antibiotic treatment, with initiation occurring more than 48 hours after infection onset, while others were administered incorrect antibiotic doses. These delays raise concerns about hospital management practices and may be attributed to prolonged diagnostic processes, such as delays in obtaining culture results and AST, as well as logistical challenges including delays in antibiotic prescribing or dispensing. Such factors can adversely impact patient outcomes and contribute to poorer disease progression (40,42,45).”

- Similar to above, what are the major prevailing reasons for delays in de-escalation or switching after the results of AST became available (noting that this occurred in almost 19%)

**Authors’ response:**

Thank you for your insightful comment. We have now outlined the major prevailing reasons for delays in de-escalation or switching after AST results became available within the 13^th^ paragraph under the **Discussion** section as follows:

“These findings reflect serious deficiencies in the timely optimization of antibiotic therapy, which may be attributed to several factors, including delays in reviewing and acting upon culture results to guide treatment adjustments, concerns about patient instability or infection severity, and clinician’s reluctance to de-escalate due to fear of treatment failure (58). Delays in de-escalation not only contradict AMS principles but may also indicate a tendency among physicians to prefer broad-spectrum antibiotics to avoid potential clinical deterioration. While this cautious approach may stem from concerns about ensuring rapid clinical recovery, it inadvertently contributes to the overuse of broad-spectrum agents, thereby increasing the risk of AMR development (58).”

- Going over the multivariable analysis in more detail and table 6 showing three independent mortality predictors:

1. Female sex (AOR=4.41)

2. DTR infection (AOR=3.11)

3. Inappropriate initial therapy (AOR=1.43)

- Did you stratify or examine the distribution of comorbidities (hypertension, CHF, etc.) by sex? Could confounders remain unrecognized (e.g., older women with multiple comorbidities)? Could ICU admission or other severity metrics modify (mediate or confound) the effect of sex on mortality?

**Authors’ response:**

Thank you for your valuable feedback and for highlighting this important issue. To address your concerns, we have conducted additional analyses to assess the potential confounding effect of sex on mortality. Specifically, we examined the distribution of comorbidities, Charlson Comorbidity Index (CCI), and ICU admission by sex. Chi-square tests were performed for categorical variables, and independent t-tests were used for continuous variables (CCI). This analysis revealed significant differences in the distribution of these factors between male and female patients, highlighting the need for their inclusion in the final multivariable model.

Based on these findings and your prior recommendations, we refined our multivariable logistic regression model to account for comorbidity burden (CCI) and ICU admission as potential confounders. After incorporating these adjustments, the previously observed association between female sex and mortality was no longer statistically significant. Consequently, we made the following amendments:

**Abstract** (at the end of the Results):

“Predictors of mortality included ‎DTR P. aeruginosa infection (adjusted odds ratio [AOR] = 2.48, 95% CI = 1.32–4.63, P < 0.01), ‎and inappropriate initial therapy (AOR = 1.40, 95% CI = 1.04–2.35, P < 0.01).”

***2.6. Statistical analysis*** (2^nd^, 3^rd^, and 4^th^ paragraphs)

“Univariate analysis was conducted to assess associations between independent variables and 30-day all-cause mortality, utilizing Pearson's chi-square (χ²) test for categorical variables and independent t-tests for continuous variables. Variables with a P-value < 0.2 in the univariate analysis were included in the multivariable logistic regression model. The variables initially included were: age group (≥ 65 years vs. 18–64 years), sex (female vs. male), site of infection acquisition (community-acquired vs. nosocomial infection), hospital department of infection acquisition (ICU vs. non-ICU), coronary artery disease, congestive heart failure, CCI, previous glucocorticoid use within the last four weeks, type of antimicrobial resistance (DTR vs. non-DTR *P. aeruginosa* infection), and appropriateness of the initial antibiotic therapy (appropriate vs. inappropriate).

Multicollinearity was assessed using variance inflation factors (VIF), with a threshold of 10 for exclusion. All VIF values were below 2.5, indicating no significant multicollinearity among the predictors. Conceptually related variables were carefully reviewed for inclusion based on clinical relevance and contribution to model fit. After careful consideration, ICU vs. non-ICU was retained for its stronger relevance to patient outcomes, while nosocomial vs. community-acquired infection was excluded. Similarly, congestive heart failure and coronary artery disease were excluded due to their conceptual overlap with CCI, which was retained as a more comprehensive measure of comorbidity burden.

The final multivariable logistic regression model was developed using a stepwise forward selection approach, which sequentially added variables based on their statistical contribution to the model. Confounding variables were retained in the model if their inclusion altered the coefficient of any significant variable by 10% or more. The glucocorticoid use, although identified in the univariate analysis (P < 0.2), was excluded during forward selection as it did not significantly contribute to the final model after accounting for other variables. The goodness-of-fit for the model was assessed using Hosmer-Lemeshow test. Adjusted odds ratios (AOR) with 95% confidence intervals and P-values were calculated for all variables in the model.”

***3.9. Mortality predictors in patients with Pseudomonas aeruginosa infections*** (2^nd^ paragraph)

“Binary logistic regression identified two main predictors significantly associated with mortality in patients with *P. aeruginosa* infection (**Table 6**). These predictors were infection with DTR *P. aeruginosa* isolates (AOR = 2.48, 95% CI = 1.32–4.63, P < 0.01), and receiving inappropriate initial antibiotic therapy (AOR = 1.40, 95% CI = 1.04–2.35, P < 0.01)”

**Table 6** has been revised to present the findings of the updated logistic regression analysis.

Please touch on these topics to increase the value of the discussion

- To what extent do isolates within your cohort demonstrate simultaneous carbapenem resistance with susceptibility to cefepime or Pip/Taz? Briefly touch on the potential mechanisms of carbapenem resistance in isolates with concomitant susceptibility to 4th gen cephalosporins or Pip/taz. Discuss whether this particular pattern is unique to your epidemiologic setting or healthcare system. This is especially relevant since you have suggested a change in empiric practices based on this data. Does this pattern of pseudomonas aeruginosa resistome generalize to other institution in your country or region? Is there any molecular regional data pertaining to oprD downregulation that you can allude to?

**Authors’ Response:**

Thank you for your insightful comment. We have now incorporated this information into:

***3.3. Infection site distribution and resistance profiles of Pseudomonas aeruginosa isolates*** (4^th^ paragraph)

“Among the CRPA isolates, 50.4% remained susceptible to ceftazidime, 48.2% to cefepime, and 40.3% to piperacillin/tazobactam.”

**Discussion** (3^rd^ paragraph)

“Nearly half of the CRPA isolates in this study exhibited concomitant susceptibility to broad-spectrum cephalosporins (e.g. cefepime, ceftazidime) and piperacillin/tazobactam. Consistent with our findings, a recent national multicenter epidemiological study conducted among 2,400 patients diagnosed with Gram-native bacteremia reported higher resistance rates of *P. aeruginosa* to carbapenems while showing lower resistance to ceftazidime and cefepime (35). This resistance phenotype is primarily attributed to the mutational inactivation or downregulation of the outer membrane porin (OprD), which selectively facilitates the uptake of carbapenems into the bacterial cell. Mutations or reduced expression of OprD decrease carbapenem influx, conferring resistance while preserving the efficacy of non-carbapenem antipseudomonal β-lactams, such as cefepime and piperacillin/tazobactam, as their uptake pathways remain unaffected (36,37). Similarly, a national study investigating the molecular mechanisms of CRPA revealed that OprD gene disruption, caused by either mutations or insertion sequence elements, was a major determinant of imipenem resistance (38). Additionally, the overexpression of efflux pumps, particularly MexAB-OprM or MexXY-OprM, contributes to this resistance profile. These efflux systems selectively expel carbapenems more efficiently than other β-lactams, leading to carbapenems resistance while maintaining susceptibility to other antipseudomonal agents (39). Further molecular epidemiological studies are needed to confirm this observed resistance pattern across different hospitals in Lebanon. These findings are crucial for optimizing empiric therapy selection, reducing reliance of last-resort antibiotics, and tailoring antimicrobial treatments based on local resistance trends.”

- I would recommend against suggesting practice changes based on observational studies in a manuscript. The more appropriate reflection based on the limitations of this study design is somewhere along the lines of ‘interesting results but need randomized data to inform practice’ etc.

**Authors’ response:**

We appreciate this insightful comment. In response, we have revised the **Discussion** section (within 9^th^ paragraph) to avoid making definitive practice recommendations based on our observational study findings. Instead, we now highlight the concerning rates of carbapenem resistance and the potential role of alternative agents while emphasizing the need for further research. The revised part reads as follows:

“Our findings suggest that carbapenem resistance among *P. aeruginosa* isolates is concerning and highlight the potential role of alternative antipseudomonal agents, such as piperacillin-tazobactam and extended-spectrum cephalosporins (e.g., ceftazidime, cefepime), which demonstrated lower resistance rates (< 16%). However, given the limitations of this observational study, further prospective studies and randomized controlled trials are needed to validate these findings and optimize empirical treatment strategies based on local resistance patterns.”

- another limitation worth mentioning along with the lack of any molecular data within this study or mention of any specific PDCs, outer porin regulation, and PBP mutations among others.

**Authors’ response:**

Thank you for highlighting this important limitation. We have now incorporated your suggestion into the ***Study limitations*** (6^th^ paragraph) as follows:

“Finally, a key limitation of this study is the absence of molecular data, which would have helped confirm the specific mechanisms underlying carbapenem and β-lactam resistance. In particular, we did not investigate factors such as Pseudomonas-derived cephalosporinases, the regulation of OprD, or mutations in penicillin-binding proteins, all of which may contribute to the observed resistance patterns. Further molecular studies are essential to identify these mechanisms and provide a deeper understanding of the resistance profile of *P. aeruginosa* isolates in our country.”

- Would any rapid diagnostic tools expedite targeted therapy in the described context of a lack of standardized antibiotic protocols and the necessity of improved stewardship?

**Authors’ response:**

Thank you for your insightful comment. We have addressed this point in the **Discussion** (19^th^ paragraph) as follows:

“The widespread use of broad-spectrum empirical antibiotics to treat MDR infections can inadvertently promote further resistance, perpetuating the AMR cycle. This study highlights the need for adopting recent technological advances, such as rapid diagnostic tools and artificial intelligence, to enhance the early and precise identification of pathogens and resistance genes. The implementation of rapid molecular-based tests, such as polymerase chain reaction and Matrix-Assisted Laser Desorption/Ionization–Time of Flight Mass Spectrometry, facilitates the prompt detection of MDR organisms, enabling timely, targeted antimicrobial therapy, and reducing reliance on broad-spectrum antibiotics (66). Additionally, Next-Generation Sequencing offers a powerful approach for comprehensive resistance profiling, enabling the identification of resistance genes, novel resistance mechanisms, and whole-genome sequencing data for epidemiological surveillance (67). Furthermore, emerging microfluidic-based diagnostic platforms offer promising solutions for rapid, real-time pathogen identification and antimicrobial resistance profiling, contributing to more precise treatment strategies. Integrating these innovations into routine clinical workflows could significantly improve antibiotic stewardship efforts, expedite targeted therapy, and minimize treatment failure and the spread of resistance. However, the widespread implementation of these advanced diagnostic tools remains limited by factors such as cost, accessibility, and laboratory infrastructure (66).”

- Within the framework of ther results showing that inappropriate initial therapy (including timing/dose errors) being common; haave you considered analyzing whether a “bundle approach” (i.e., checklists for sepsis, analysis of DTR risk factors/daily stewardship rounds) could systematically lower this rate?

**Authors’ Response:**

Thank you for your insightful comment. We acknowledge that a bundle approach, incorporating sepsis checklists, analysis of DTR risk factors, and daily antimicrobial stewardship rounds, has the potential to improve the appropriateness of initial antibiotic therapy and reduce treatment delays. However, our study primarily focused on assessing the impact of inappropriate initial therapy on mortality rather than evaluating specific interventions to mitigate this issue. We agree that implementing a structured bundle approach could systematically lower the rate of inappropriate initial therapy, and this represents an important avenue for future research. To address this, we have now expanded the **Discussion** section (8^th^ paragraph) to highlight the need for such interventions and conducting further studies to evaluate their effectiveness in improving antibiotic selection and patient outcomes, as follows:

“Several evidence-based strategies have been proven to optimize antibiotic use in clinical practice while minimizing the risk of inappropriate initial therapy. Among these, care bundles play a crucial role in guiding empirical antibiotic selection, supporting clinical decision-making, and improving prescribing practices. These bundles incorporate key elements such as novel rapid microbiological diagnostics, the use of inflammatory markers to determine the need for antibiotics, identification of patient-specific risk factors for DTR infections, and personalized antibiotic therapy, including dose optimization (46,47). Additionally, standardized protocols such as early recognition tools and sepsis checklists are essential in ensuring timely and appropriate antibiotic administration (48). The implementation of a multidisciplinary approach, including AMS strategies such as preauthorization of restricted antibiotics, expert consultation, and routine AMS rounds to review ongoing antibiotic therapies, has also been associated with improved antibiotic utilization and patient outcomes (49). Future national studies are needed to evaluate the implementation of these interventions and their effectiveness in reducing the incidence of inappropriate initial therapy.”

- Please ascertain within your cohort the instances of inappropriate antibiotic therapy (with Pip/Taz or cefepime) where a carbapenem should have been prescribed empirically (based on risk factors).

**Authors’ Response:**

Thank you for this insightful comment. We acknowledge that assessing the appropriateness of empirical therapy based on risk factors for MDR *P. aeruginosa* is a crucial aspect of antimicrobial stewardship. However, our study did not specifically investigate the risk factors that would necessitate the empirical use of carbapenems over piperacillin/tazobactam or cefepime. Key risk factors such as prior invasive medical procedures, recent surgeries, and residence in long-term care facilities, were not systematically documented in the medical records, limiting our ability to analyze their impact.

Instead, our assessment of inappropriate initial antibiotic therapy was based on predefined criteria, as **already mentioned** in 4^th^ paragraph of section 2.4 under the Methodology section:

“The appropriateness of the initial antibiotic therapy was assessed by determining whether one or more antibiotic agents were administered within 48 hours of infection onset, and whether the causative pathogen was susceptible to these agents according to AST results. The antibiotics must have been delivered via the correct route and at an appropriate dosage regimen. Any initial antibiotic therapy that did not meet these criteria was considered inappropriate (20).”

- Suggest emphasizing in the discussion any missed covariates (e.g., baseline severity scores, or comorbid conditions) that might influence results.

**Authors’ response:**

Thank you for your insightful suggestion. We have addressed the potential impact of missed covariates in the ***Study limitations*** (within 2^nd^ paragraph) as follows:

“Second, the retrospective design and reliance on medical records for data collection present inherent limitations. Critical information that may not have been documented could have been omitted, potentially affecting the completeness of the analysis. The absence of key data restricts our ability to establish causal relationships, and the inability to account for certain confounding variables may hinder our capacity to accurately identify mortality predictors associated with DTR *P. aeruginosa* infections. For example, essential variables such as admitting diagnoses, laboratory results, follow-up cultures, clinical responses to treatment, and infection recurrence were not consistently retrieved from medical records. The lack of this information limits the depth of our analysis and introduces potential bias when evaluating factors that influence patient outcomes. Furthermore, although we accounted for comorbidities using the CCI, we were unable to include baseline severity scores (e.g., APACHE II, SOFA) due to incomplete documentation. These scores could have provided additional insights into the impact of disease severity on patient outcomes. The absence of follow-up cultures specifically restricted our ability to assess how resistance patterns may have evolved during treatment.”

- AST data from Lebanon would be interesting to a global audience. If you are able to provide raw AST data on all isolates in a supplement, it would be appreciated and carry value.

**Authors’ response:**

Thank you for your valuable suggestion. We have included the raw antimicrobial susceptibility testing (AST) data for all isolates in the ***supplementary appendix***, as per your request, to provide valuable data for the global audience interested in regional resistance patterns. Additionally, we have cited the supplementary appendix in the relevant results section of the manuscript to ensure accessibility of this data.

6. PLOS authors have the option to publish the peer review history of their article ([what does this mean?](https://eur01.safelinks.protection.outlook.com/?url=https%3A%2F%2Ftrack.editorialmanager.com%2FCL0%2Fhttps%3A%252F%252Fjournals.plos.org%252Fplosone%252Fs%252Feditorial-and-peer-review-process%2523loc-peer-review-history%2F1%2F010f01940762ca77-e74db438-7e1e-4d63-9582-b91c07061862-000000%2FTmttHnTgVPmNcat0jBryG6lkSOvloN9YWvB1NhstlPg%3D190&data=05%7C02%7Cr.itani%40bau.edu.lb%7C0c5827a011fc49ba6b3408dd26567c04%7Cae73320887834b9394a5c5f29fb71fa9%7C0%7C0%7C638708875551783663%7CUnknown%7CTWFpbGZsb3d8eyJFbXB0eU1hcGkiOnRydWUsIlYiOiIwLjAuMDAwMCIsIlAiOiJXaW4zMiIsIkFOIjoiTWFpbCIsIldUIjoyfQ%3D%3D%7C0%7C%7C%7C&sdata=V7FB0PMzkCZgotz%2Faimje1cknLQjl1v4HpUjRqeFKEk%3D&reserved=0)). If published, this will include your full peer review and any attached files.

If you choose “no”, your identity will remain anonymous but your review may still be made public.

**Do you want your identity to be public for this peer review?** For information about this choice, including consent withdrawal, please see our [Privacy Policy](https://eur01.safelinks.protection.outlook.com/?url=https%3A%2F%2Ftrack.editorialmanager.com%2FCL0%2Fhttps%3A%252F%252Fwww.plos.org%252Fprivacy-policy%2F1%2F010f01940762ca77-e74db438-7e1e-4d63-9582-b91c07061862-000000%2FgHtV6aO1OsbKMHe1P3llzD29wlNJRmkHSsBG3ELRMNI%3D190&data=05%7C02%7Cr.itani%40bau.edu.lb%7C0c5827a011fc49ba6b3408dd26567c04%7Cae73320887834b9394a5c5f29fb71fa9%7C0%7C0%7C638708875551796437%7CUnknown%7CTWFpbGZsb3d8eyJFbXB0eU1hcGkiOnRydWUsIlYiOiIwLjAuMDAwMCIsIlAiOiJXaW4zMiIsIkFOIjoiTWFpbCIsIldUIjoyfQ%3D%3D%7C0%7C%7C%7C&sdata=EXyqd4B9YFuX%2Fz%2FBls1Seq%2Bal6hunt2EKDnqXK9GIb4%3D&reserved=0).

Reviewer #1: **Yes:** Mohamad Yasmin
